# DNA Profiling in Human Identification: From Past to Present

**DOI:** 10.21315/mjms2023.30.6.2

**Published:** 2023-12-19

**Authors:** Sundararajulu Panneerchelvam, Mohd Nor Norazmi

**Affiliations:** 1 School of Health Sciences, Universiti Sains Malaysia, Kelantan, Malaysia; 2 Malaysia Genome and Vaccine Institute, National Institutes of Biotechnology Malaysia, Selangor, Malaysia

**Keywords:** chromosome, DNA typing, likelihood ratio, microsatellite, minisatellite, mitochondria, match probability, polymorphism

## Abstract

Forensic DNA typing has been widely accepted in the courts all over the world. This is because DNA profiling is a very powerful tool to identify individuals on the basis of their unique genetic makeup. DNA evidence is capable of not only identifying the presence of specific biospecimens in a crime scene, but it is also used to exonerate suspects who are innocent of a crime. Technological advancements in DNA profiling, including the development of validated kits and statistical methods have made this tool to be more precise in forensic investigations. Therefore, validated combined DNA index system (CODIS) short tandem repeats (STRs) kits which require very small amount of DNA, coupled with real-time polymerase chain reaction (PCR) and the statistical strengths are used routinely to identify human remains, establish paternity or to match suspected crime scene biospecimens. The road to modern DNA profiling has been long, and it has taken scientists decades of work and fine tuning to develop highly accurate testing and analyses that are used today. This review will discuss the various DNA polymorphisms and their utility in human identity testing.

## Introduction

**Figure 1:**
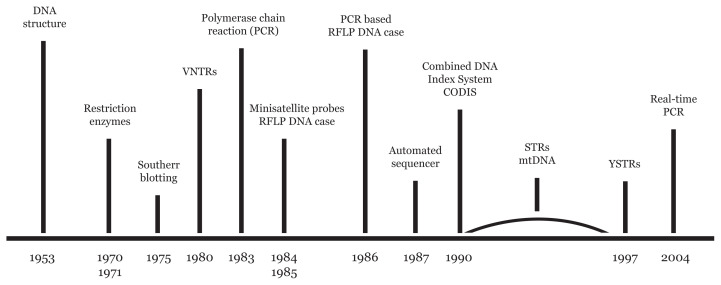
Development in DNA techniques that facilitated the development of DNA profiling. Since the discovery of the structure of DNA, various developments in techniques to characterise DNA have facilitated the discovery of DNA profiling methods

The role of forensic scientists is to analyse physical/biological trace evidence from a crime scene, victim or suspect using available tools and technologies, to enable reconstruction of a situation of interest. For decades, blood group and protein markers have been used in the identification and individualisation of human biospecimens (1, 2). However, the nature of blood and protein markers is unduly compromised by the environment. In most cases, they are helpful to exclude rather than to include the alleged suspects. The advent of DNA fingerprinting and its application in 1986 ushered in a new approach in processing biological trace evidences such as blood, semen, tissue, teeth and bones (3–5). The time line shown in Figure 1 charts the efforts of various discoveries ultimately culminating in the sophistication of the current DNA profiling techniques used routinely. A reliable (standardised and consistent) method of human identification in criminal investigation is essential to ensure justice is upheld. Coupled with robust statistical analyses and database, the ability to distinguish one person from another with great certainty provide high confidence in establishing individualisation. DNA database can also help determine previous record of the suspect’s involvement with previous crimes; and to verify or negate the involvement of individuals charged with the crime. It should be noted that DNA profiling is not a standalone tool and should be analysed together with other important information obtained during the occurrence of a crime.

### DNA

#### DNA Structure

DNA, the repository of all genetic traits of a human, is present in all nucleated cells except red blood cells in the form of a double helix structure tightly bound with histone proteins in chromosomes. DNA is unchangeable from cell to cell within an individual and contains all the genetic information necessary (6, 7). In humans, there are 23 pairs of chromosomes—22 pairs of autosomal chromosomes and a pair of sex chromosomes—XX in females and XY in males (8). The DNA double helix consists of two polynucleotide chains comprising nucleotide monomers. Each nucleotide consists of a sugar (deoxyribose), a phosphate group, and a nitrogen-containing base, namely, adenine (A) thymine (T) guanine (G) or cytosine (C). Adenine always bonds with its complementary base, thymine, whereas cytosine always bonds with its complementary base, guanine. Hydrogen bonds between complementary bases hold together the two polynucleotide chains of DNA. A and G have a two-ring structure. C and T have just one ring. The distance between the two chains is kept constant by the base pairs (bp). This maintains the uniform shape of the DNA double helix. These bp (A-T or G-C) stick into the middle of the double helix, forming the rungs of the double helical spiral ladder (9).

DNA replication is the process in which DNA is copied. It occurs during the synthesis (S) phase of the eukaryotic cell cycle. DNA replication is a semi-conservative process initiated at specific point known as origin. It is a bidirectional process in which each strand of the double helix acts as a template. Strand separation is accomplished by helicase enzyme and the building of new complementary strand with complementary bases is accomplished by DNA polymerase with high fidelity (6, 9). The two daughter molecules that are produced contain one strand from the parent molecule and one new strand that is complementary to it. As a result, the two daughter strands are both identical to the parent strands. DNA replication is thus a semi-conservative process since half of the parent DNA molecule is conserved in each of the replication cycle. Importantly, the DNA sequence is copied essentially unchanged into all daughter cells, providing an ‘exact match’ in the DNA profile obtained from various biospecimens of the same individual.

In density gradient centrifugation, DNA in solution exhibits two bands—one is denoted as the major band and a minor satellite band. There are 3 billion bp found distributed along the length of the 23 pairs of human chromosomes coding for approximately 30,000 genes (10). Segments of DNA that code for a specific protein are known as gene. These genes occupy only 1%–2% of the 3 billion bp and these gene specific sequences are known as coding regions. The remaining 98%–99% of human genome is made up of non-coding DNA (6, 7). An allele is a modified or alternative form of a gene that is located on a specified position on a specific chromosome. Coding sequences are frequently interspersed with non-coding repeat sequences. Noncoding regions generally contain DNA sequences that may be either of a single copy or exist as multiple copies called repetitive DNA. Besides nuclear genome there are multiple copies of mitochondrial genome present in each of the human cell which are also used in forensic human identification (6).

#### DNA Polymorphism

Polymorphism in genetics means a trait that differs among individuals (6, 11) and the term variation is frequently interchangeably used to denote a polymorphic trait. Prior to the introduction of DNA typing, blood groups, serum and salivary protein variation among individuals were used for identification/individualisation of human biospecimens. Variation in proteins is the manifestation of the variation in DNA. Variation in DNA sequence among individuals is termed as DNA polymorphism and is heritable. Variation in DNA sequence is due to mutation, which may arise due to replication error or induced by external agents such as radiation or chemical mutagens. Such new mutations in coding and noncoding sequences accumulate over a period of time and in consequence, no two humans share the same genome except identical twins who will share identical DNA. Hence DNA polymorphism is a DNA sequence variation that is not associated with any observable phenotypic variation and can exist anywhere in the genome, not necessarily in a gene. Hence DNA polymorphism means one of two or more alternative forms (alleles) of a chromosomal region that either has a different nucleotide sequence or it has variable numbers of tandemly repeated nucleotides (6, 11). DNA polymorphism in human genome include single nucleotide polymorphisms (SNPs), variable number of tandem repeats (VNTRs), transposable elements (e.g. Alu repeats), structural alterations and copy number variations (6).

#### DNA Polymorphic Genetic Markers in Human Identification

##### Single nucleotide polymorphisms

SNP is a single bp change, a point mutation, and the site is referred to as SNP locus. SNPs are base substitutions, insertions or deletions occurring at single positions in the genome of any organism. SNPs are the most common type of DNA variation, occurring with a frequency of one in 350 bp and accounting for more than 90% of DNA sequence variation (11). Most SNPs are found to be present in the non-coding regions of the genome. Due to their abundance, SNPs have become important genetic markers for mapping human diseases, population genetics and evolutionary studies (12). SNPs have become very important since technologies for DNA sequencing have become feasible and widely available (13, 14). Lack of recombination make mitochondria and Y chromosome SNPs suitable as lineage markers and kinship testing in missing-persons cases (12).

##### Restriction fragment length polymorphisms

Bacteria are endowed with special molecular scissors known as endonucleases capable of cutting foreign DNA having specific recognition sites for that endonuclease. Restriction enzymes are site-specific DNAses that cleave a DNA molecule whenever the specific sequence is recognised, which is usually a 4–6 base palindrome. Each bacterium has its own unique restriction enzyme. Each endonuclease recognises only one type of sequence in the foreign DNA. Restriction enzyme recognition sites may be present in some genomes and absent in others. A specific endonuclease used to cut DNA of an organism having restriction sites for that enzyme will result in fragments differing in their length due to the different location of recognition sites along the length of the DNA of the organism (15, 16). Two individuals belonging to the same species will differ in the number of restricted fragments and exhibit uniqueness. Due to the enzyme’s sequence specificity, digestion of a particular DNA results in a reproducible array of restriction fragment length polymorphisms (RFLPs) or length differences in homologous fragments between different DNA samples. These length differences can be the result of a point mutation obliterating an existing restriction site or generating new restriction site. Insertion or deletion of DNA between two restriction enzyme cut sites also result in new RFLP.

RFLP was one of the earlier profiling methods used for individualisation. However, the technique requires large amounts of DNA and is rather cumbersome to perform.

##### Tandem repeats

Satellite DNA is mainly present in heterochromatin or the tightly packed regions of chromosomes in centromeres, telomeres and sometimes even in the euchromatin region (active region of the genome). Satellite DNA consists of arrays of tandem repeats or repeats arranged side-by-side in a head-to-tail fashion. These repeats can be as small as 1 bp−6 bp long or it may be 9 bp−100 bp long. The short tandem repeats (STRs) (1 bp–6 bp long) are known as microsatellite or simple sequence repeats (SSRs), while the longer tandem repeats (9 bp–100 bp) are called minisatellites or variable number tandem repeats (VNTRs) (17–20).

The regions in between two simple sequence repeats or microsatellite are termed as ‘inter simple sequence repeats’ or ISSRs. Based on the length of the repeat motif, STRs are classified into mono-, di-, tri-, tetra-, penta- and hexanucleotide repeats. Dinucleotide repeats are the most abundant in the human genome. The short sequence of DNA stretches in satellite DNA varies from individual to individual. In forensic case work, tetra and penta nucleotide repeats are chosen and validated for use. STRs have higher mutation rate than SNPs. The mutation rate for SNPs is approximately 10^−9^ per generation and for STRs it is 10^−6^ to 10^−2^ per generation. Three possible mechanisms for the high STR mutation rate have been suggested. They are (i) unequal crossing over in meiosis, (ii) retro-transposition mechanism and (iii) strand slippage replication. It is suggested that STR mutation rate increases proportionally with repeat number (21, 22).

**Figure 2:**
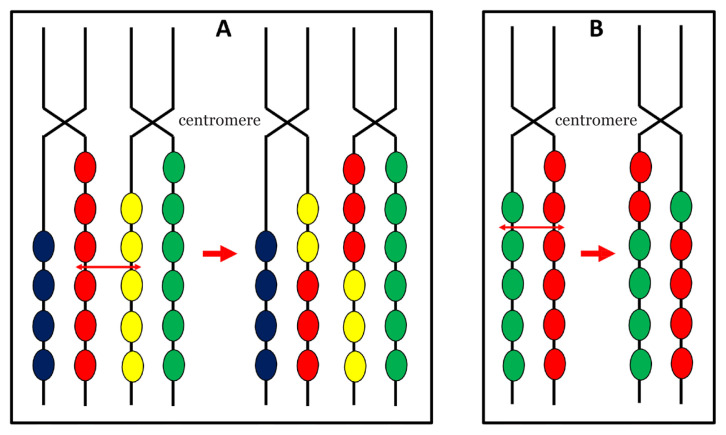
Unequal cross over during meiosis

Unequal crossing over between homologous pairs of chromosomes in satellite DNA in meiotic prophase results in addition or reduction in the number of repeat units (Figure 2A). Sister chromatid exchanges in meiotic prophase also leads to increase or decrease of repeat units (Figure 2B).

Retrotransposition mechanism suggest that STRs are generated by retrotranscripts. Further studies are required to elucidate the process.

**Figure 3:**
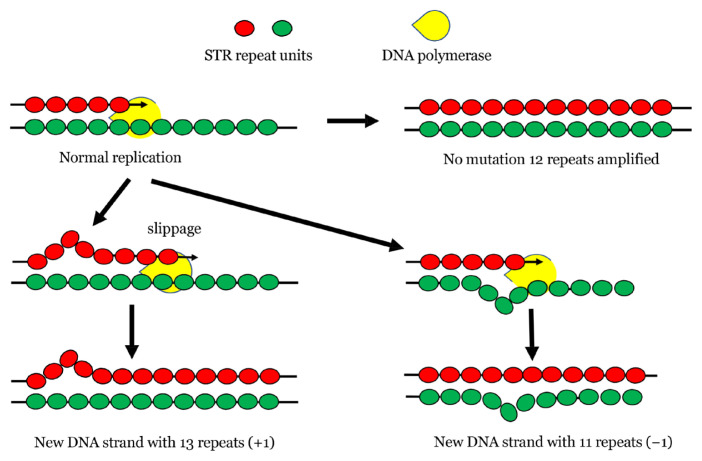
Strand-slippage replication resulting in addition or reduction in STR number. Also known as DNA slippage or slipped strand mispairing or DNA polymerase slippage. Most studies favour slippage as the main cause for STR mutation process. The mispairing in the region of repeat units between template DNA and the newly synthesised DNA may occur during DNA replication resulting in the addition or reduction of STR number

Strand-slippage replication is also known as DNA slippage or slipped strand mispairing or DNA polymerase slippage. Most studies favour slippage as the main cause for STR mutation process. The mispairing in the region of repeat units between template DNA and the newly synthesised DNA occurs during DNA replication (Figure 3) resulting in addition or reduction of STR number.

In 1993, the Commission of the International Society of Forensic Haemogenetics (ISFH) proposed the nomenclature method of STRs.

D7S820 is one of the common STRs used in DNA profiling. The D stands for DNA and the number following D indicates the chromosome on which the STR is located. S is for the STR sequence and 820 (in D7S820) is the unique sequence number (23). The STR alleles are named according to the number of repeat units it contains. The STRs are inherited in a simple Mendelian fashion. An individual inherits an allele each from his/her father and mother. A person may be homozygous (the same allele on the maternal and the paternal chromosomes) or heterozygous (different alleles on the two chromosomes). STRs used in forensic human identification are from autosomal (autosomal STRs), Y (Y STRs) and X (X STRs) chromosomes (24–30). Autosomal STRs used in DNA profiling are single locus multiple allelic markers used for identification and individualisation of forensic biospecimens and for ascertaining parentage in paternity cases.

The X and Y chromosomes are inherited in unique ways depending on the sex. The X chromosome consists of hundreds of genes. The Y chromosome contains sex determining region (SRY) gene and most of the Y chromosome is made of noncoding DNA. In males, the X and Y recombine at the pseudo autosomal region (PAR region), hence, the greater part of X and Y chromosome is inherited without recombination in meiosis (31, 32). The X chromosome is in pairs in females, and they undergo extensive recombination and inherited. The X or Y chromosome from a male is inherited to his daughter or son, respectively, without recombination. While the X chromosome from females are inherited to both sexes with recombination. The lack of recombination makes Y STRs to be inherited as a haplotype and all male descendants from a male lineage share the same STRs. The Y STRs are more frequently used in rape cases. Y STRs are more useful in gang rape cases to identify male individuals from the mixed biospecimen from females.

A robust database is available for the distribution of Y STRs haplotypes and for Y SNPs for various populations and provides forensic investigators with clues to the source of the male genetic material left at a crime scene (YHRD) (33). Haplotype frequency data for X chromosome STRs is available in ChrX-STR. org 2.0 (34). This database covers many issues concerning the usage of X-chromosomal markers for forensic purpose. X STRs are more useful as supplementary data for discrimination of individuals in incest cases.

##### Mitochondrial DNA

Mitochondria are cytoplasmic organelles present in multiple copies ranging from 500–1,000 in human cells. Each mitochondrion contains extra chromosomal circular DNA devoid of bound histone proteins (35, 36). The complete sequence of human mitochondrial DNA (mtDNA) genome was ascertained and it is known as the Cambridge Reference Sequence (CRS). The mtDNA of humans is about 16,569 bp (37, 38). The two strands of the circular mtDNA differ in densities due to base composition and they are referred as heavy and light chains. The heavy chain contains more genes compared to the light chain. The mtDNA genes of humans are devoid of introns. The origin of replication is present in the control region which is about 1.2 kb in length, which is the noncoding part of the mtDNA consisting of highly polymorphic hypervariable (HV) regions. This stretch of noncoding sequence consists of the hypervariable HVI, HVII and HVIII regions (39). The mtDNA does not have DNA repair mechanisms and as a consequence has high mutation rate compared to nuclear genome. mtDNA is passed from one generation to the next, essentially unchanged, solely through the maternal line of a family. During fertilisation of an egg the male sperm does not typically contribute mitochondria to the offspring. Hence there is only one mtDNA genotype (that of the mother) in an individual and all mitochondrial genomes are approximately genetically identical, and the condition is known as homoplasmy. In many mitochondrial diseases, however, since the number of copies of mitochondria within a particular cell is high, wild-type and mutant maternal alleles coexist, and this is known as heteroplasmy (40, 41).

### DNA Profiling Techniques

DNA profiling is also known as DNA typing, DNA fingerprinting, genetic fingerprinting, genotyping or identity testing. However, the term DNA profiling is more preferred since a wide range of tests are carried out by DNA sequencing with improved technology. The DNA profiling technique has evolved in three phases. First was the use of multi-locus VNTR probe-based RFLP developed by Sir Alec Jeffreys (42). The second phase utilised single locus probe (SLP)-based RFLP. Finally, the third phase, which is the present DNA profiling technique uses STR. All DNA profiling methods involve four basic strategies that include extraction of DNA from the source biospecimen, quantification, electrophoresis and detection.

#### RFLP Using MLP/SLP Probes (Phase 1 and 2)

**Figure 4:**
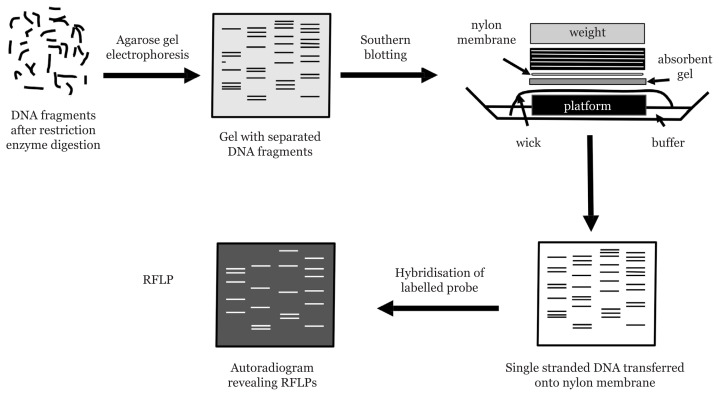
Identification of VNTR using a labelled MLP

The original DNA profiling developed by Sir Alec Jeffreys involved the use of a multi-locus probe (MLP) against the VNTR across the human chromosomes (3, 4, 42). High molecular weight DNA extracted was subjected to restriction enzyme digestion. The restricted DNA was then separated in agarose gels using electrophoresis in the presence of a radioactive labelled DNA marker. Electrophoresis separates the restricted fragments towards the positive end depending on their size. Smaller fragments which move faster will be at the bottom of the gel and the longer fragments which move slower are closer towards the negative end. Double stranded DNA fragments in the gel were then converted to single strands by denaturation before being transferred to a nylon membrane for autoradiography (43). Radioactive labelled MLP was then used to hybridise to the single stranded DNA fragments on the nylon membrane. Then the nylon membrane was exposed to an X-ray film. The various steps in RFLP technique is shown in Figure 4. Dark bands appear on the X-ray film due to the binding of radioactive labelled probe with the complementary VNTR carrying fragments. The band patterns obtained are unique to an individual where discrete bands of between 6 and 8 bases are enough to distinguish between different individuals. The first application of DNA profiling in forensic identification was in 1985 in a case that exemplifies the power of DNA evidence to link crime-scenes, to exclude suspects and to support convictions.

In that particular case, a suspect was arrested for allegedly raping and murdering two minor girls in a UK suburb. The suspect was arrested based on suspicion. DNA profiling using MLP on a sample of semen left in the crime scene demonstrated that a man had been responsible for both crimes. But the profile did not match with the arrested person who was subsequently released. Police decided to flush out the killer by taking blood samples from adult males residing in the surrounding villages. After testing 4,852 male samples, the 4853rd sample tested matched the semen DNA profile. The suspect was arrested and subsequently sentenced (3).

The technique was met with almost universal acceptance and admissibility in court. Defence attorneys struggled unsuccessfully to demonstrate the potential flaws and setbacks of the early technology. During the first string of cases, law enforcement agencies sent evidence samples to private biotechnology companies for analysis. At the same time, defence attorneys struggled to combat DNA-based evidence in court because the technology used by the private companies was strongly backed by scientific methods.

Meanwhile the US Federal Bureau of Investigation (FBI) assumed a role of standardising the technique of DNA fingerprinting in forensic DNA analysis and attempted to standardise most of the methods and procedures. Controversies led to new concerns regarding laboratory error and regulation (44, 45).

Further, DNA analyses with RFLP required comparatively large amount of high molecular weight DNA. Degraded samples could not be examined with precision. A single band is sometimes ambiguous, because it may be from a homozygote or from a heterozygote. The small differences between adjacent alleles necessitated grouping them into ‘bins’, which complicated the statistical analyses. As an alternative to MLP/RFLP, many single locus VNTRs (17) were recognised and single locus probes (SLPs) were constructed so the DNA strands only bind with the alleles at one specific locus. The band patterns produced by SLP technology are easier to read and the results also allowed for more precise frequency statistics because data can be collected regarding how often different alleles occur at these specific locations among different populations. To produce significantly individualised results, multiple SLP tests have to be performed. Six genetic loci namely D1S7, D2S44, D4S139, D10S28, D14S13 and D17S79 were recommended for use. However, VNTR testing was still problematic. The technique was not very efficient, gel electrophoresis measurements were messy and far from exact, and large amount of DNA samples were needed (45). Forensic DNA samples frequently are degraded or are collected post-mortem, which means that they are of lower quality and prone to provide less-reliable results than samples that are obtained from a living individual. Some of the concerns with DNA profiling, and specifically the use of RFLP, subsided with the development of polymerase chain reaction (PCR) and STRbased profiling which, is now the primary technique used, to replace the RFLP-based on MLP/SLP method of DNA profiling.

#### Polymerase Chain Reaction

The next advancement of DNA typing technology started with the discovery of PCR (46). This revolutionary technology enabled scientists to amplify a specific segment of DNA in a machine designed to carry out many cycles of replication providing a large quantity required for analysis. PCR is an in vitro exponential amplification of a specific segment of DNA from a small amount of DNA, typically less than 1 mg (47). There are four important components in the PCR process: i) the DNA template, ii) oligonucleotide primers, iii) DNA polymerase and iv) deoxynucleotide triphosphates (dNTPs). The DNA template consists of the target DNA sequence to be amplified whilst the oligonucleotide primers are short single stranded DNA molecules that bind with complementary base pairing to opposite strands of the template DNA at either end of the sequence to be amplified (46, 47). The DNA polymerase is an enzyme that copies the target sequence (33). Taq polymerase is the commonly used DNA polymerase as it is thermostable at high temperatures, which can effectively copy the original template DNA by extending the primers and making complementary copies of the original template DNA. dNTPs refer to the four bases present in DNA (A, G, T and C) which are used by the DNA polymerase as building blocks to synthesise new DNA strands. The target DNA is amplified over several cycles. Each cycle has three stages carried out at different temperatures—consisting of denaturation, annealing and extension of the target DNA in a single cycle.

#### STRs Typing Technique

##### Autosomal STR analysis

Presently, PCR-based autosomal STRs, is the technique used routinely in forensic labs for identity and individualisation. The autosomal STRs chosen for DNA profiling comprises tetra and penta core repeat STRs with no linkage located on different sets of chromosomes (48, 49). The primer sets for amplification of various STRs are designed to facilitate multiplexing of primers, which means that several primer sets can be used to amplify multiple target STRs at the same time. The PCR STR method essentially involves amplification of STRs using primers in a PCR machine and separation of the fragments corresponding to allelic ladders and detection of the fragments (50, 51). The first commercial kits were triplex STR kits using polyacrylamide gel electrophoresis (PAGE) for separation of fragments carrying STRs alleles, which were then detected with silver stain or fluorescence.

**Figure 5:**
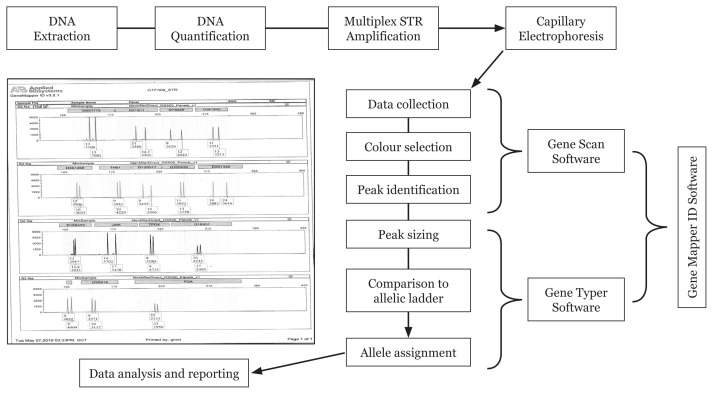
General principle of the STR technique for DNA profiling. Unknown DNA samples are extracted and quantified before being amplified by PCR using specific STR primers labelled with different fluorophores. The amplified DNA samples are then electrophoresed in capillaries containing polymer to separate the labelled DNA according to size in an automated fragment analyser/gene sequencing machine. The fluorescently tagged DNA molecules are detected and the software in the genetic analyser translates fluorescence intensity data into electropherograms against a set of allelic ladders as reference. The software then labels peaks that meet certain criteria with allelic designations and the resulting electropherogram contains genotype of the individual/biospecimen for the tested STRs

In 1998, the FBI developed the combined DNA index system (CODIS) data for 13 STRs which are stored data of DNA profiles obtained from convicted persons and unidentified DNA profiles from crime scene biospecimens. These STRs are known as CODIS STRs (50). Later, commercial kit multiplexing primers for 15 STRs and amelogenin gene for sex determination, were made available which further enhanced the CODIS STR database (52). STR primers are labelled with different fluorophores. The kits contain allelic ladder and internal size ladder. The amplified unknown DNA samples with these kits were electrophoresed in capillaries in an automated fragment analyser/gene sequencing machine. Capillary electrophoresis (CE) is an electrophoretic separation within a small diameter capillary, a long tube made of fused silica, typically 50 μ internal diameter that is filled with a sieving medium. The sieving medium is typically an entangled polymer that is designed to separate DNA fragments based on size. The multiplex/multicolour PCR products for each sample are combined with an internal size standard (DNA fragments of known size labelled with a different dye colour), heat denatured, then placed in a 96-well tray on the CE instrument. Electrophoresis through the sieving medium in the capillary separates the DNA fragments by size. A laser continuously illuminates a detection window that is located near the end of the capillary. The fluorescently tagged DNA molecules are detected during electrophoresis as they reach this laser detection window. Smaller DNA molecules reach the window before larger molecules (50–53). The software in the genetic analyser translates fluorescence intensity data into electropherograms and then labels any detected peaks with such descriptors as size in nucleotides and peak height in relative fluorescence units or RFU. Using allelic ladders as reference, the software then labels peaks that meet certain criteria with allelic designations and the resulting electropherogram contains genotype of the individual/biospecimen for the tested STRs. The steps involved in STRs-based DNA profiling are shown in Figure 5. The kits are so sensitive and require only about 1 ng of DNA sample for amplification. To date, although most laboratories use the 15 STRs kits, assays that measure 23 or more STRs are now available, increasing the discrimination probability of STR DNA profiling. Autosomal STR-based DNA profiling is now routinely used in processing biospecimens from crime scene and in parentage testing.

##### Sex-chromosome STRs

Commercial kits are also available for amplification of Y and X STRs. The method of analysis is the same as that of autosomal STRs. The Y STRs and X STRs are routinely used in gang rape and incest cases to provide supportive evidence and for greater discrimination in addition to autosomal STRs (54–56).

##### Population database and interpretation of STRs profiling data

The STR profile obtained from a crime scene sample may provide three interpretations when compared to that of a suspect: i) Exclusion: the sample profile did not match with the suspect’s profile; ii) Inclusion: the STR profile from the crime scene specimen matches with that of the suspect’s STR profile and iii) Inconclusive: no decision possible. The DNA typing data from a crime scene provides the genotype comprising two alleles for each of the STRs tested—one from the maternal chromosome and the other from the paternal chromosome.

Exclusion does not require any statistics but inclusion requires statistics to provide verbal predicate of how reliable the match is. Random match probability (RMP) is the statistical approach used to assess that the DNA profile is unique and that it is unreasonable that another person in the population might have the same STR profile. The RMP is calculated applying product rule of genotype frequencies of alleles in the population to which the suspect belongs. Hence RMP is dependent on the distribution of allele frequencies in a population which is in Hardy-Weinberg equilibrium (HWE). HWE is the foundation for population genetics and explains how populations continue unchanged in allele frequencies (and thus phenotype) unless acted on by one of the four evolutionary forces namely mutation, selection, gene flow (or admixture) and drift. Usually, a sample size of greater than 100 samples is sufficient to make reliable projections about a genotype’s frequency in the larger population (57, 58). Published STR allele frequency distribution of various populations is available in STR base (59).

It would be appropriate to explain the calculation of RMP with an illustration. Consider a case in which the bloodstain from a crime scene was DNA profiled for three STRs. Two individuals were arrested on suspicion in that case. Biospecimens from the two were tested for the same three STRs. The hypothetical case data is provided in Table 1.

The expected genotype frequencies of a population in HW for two alleles p and q are equal to the proportion: p^2^ + 2pq + q^2^. Applying this rule, the frequencies of the alleles are calculated and the RMP is calculated following the product rule that is, multiplying the genotype frequencies as shown in Table 1.

The RMP tells how likely it is that a random person’s DNA profile will match the crime sample. To take the match probability into account one has to calculate the likelihood ratio (LR).


$$\text{LR}=\frac{\begin{array}{c} \text{Probability of observing the evidence}\\ \text{if the defendant is guilty} \end{array}}{\begin{array}{c} \text{Probability of observing the evidence}\\ \text{if the defendant is innocent} \end{array}}$$

The LR measures the impact of the evidence. For the case in Table 1, suspect 2 was guilty then his DNA profile would match the crime sample, and so the probability of observing this evidence is 1. If suspect 2 was not the culprit, then the probability of observing the evidence (the denominator in the LR) is just the random match probability. So, the LR is simply the reciprocal of RMP. The LR measures the impact of the evidence.

In paternity, testing LR is also known as paternity index (PI) which is the ratio of the chance that the alleged father (AF) would transmit the obligate allele to that of the chance that some other man of the same race could have transmitted the allele (population frequency). The PI for each genotype is calculated by multiplying individual PIs to obtain the combined PI (CPI). The CPI is a measure of the strength of the genetic evidence. It indicates whether the evidence fits better with the hypothesis that the tested man is the father or with the hypothesis that someone else is the father. Then the genetic evidence is converted to probability of paternity (POP) using Bayesian theorem. The CPI is used in the Bayes formula along with another variable called a prior probability (PP). This variable represents the social evidence. Testing labs typically use a value of 0.5 for the PP assuming this is a neutral, unbiased value. The Bayesian formula is CPI / CPI + (1 − PP) × 100. The statistics for paternity calculation is illustrated with a hypothetical paternity test case for three STRs in Table 2.


$$\text{LR}=\text{PI}=\frac{\begin{array}{c} \text{Chance of alleged father }(\text{X})\\ \text{contributing the obligate allele} \end{array}}{\begin{array}{c} \text{Chance of random man }(\text{Y})\\ \text{contributing the allele} \end{array}}$$

Where,

Alleged father (AF) = X = 1 if homozygous; 0.5 if heterozygous;
Random man = Y = allele frequency

##### Low copy number DNA typing

The potential of STR-based DNA typing is widely accepted in various courts. Tabereit et al. (60) published genotyping of samples with very low DNA quantities using PCR. The sensitivity of routine STR typing requires 0.5 ng–2 ng of DNA template and 1 ng being the recommended quantity in many kits. The sensitivity of STR typing using second generation multiplex (SGM) kits is as low as 100 pg. The low copy number (LCN)-based STR typing involves mainly increasing the number of PCR cycles from normal 28 to 34 and above. LCN method is used to analyse DNA from charred human remains and minute amounts of blood. LCN typing has greater potential for errors in amplification. It is also suggested that the LCN results are not reproducible, which undermines the probative value of the results. LCN is mainly used in analysing ancient samples from decomposed bones by anthropologists and forensic scientists. The LCN typing is also known as trace DNA typing and touch DNA typing (60–64).

##### Combined DNA index system

CODIS blends computer and DNA technologies into an effective tool for fighting violent crimes. The current version of CODIS uses two indices to generate investigative leads in crimes where biological evidence is recovered from the crime scene. The convicted offender index contains DNA profiles of individuals convicted of theft, sex offences and other violent crimes. The Forensic index contains DNA profiles developed from crime scene evidence. CODIS utilise computer software to automatically search these indexes for matching DNA profiles. CODIS does not store criminal history information, case-related information, social security numbers or date of birth. DNA examiners use CODIS software and transfer unknown subject profiles into forensic index, where they are searched against other unknown subject profiles. Forensic profile developed at local laboratories is also searched against the convicted offender index. A match known as ‘cold hit’ provides the police with an investigative lead. To begin with, the FBI included 13 STRs as core CODIS STRs so all the data compiled in the two indexes had the genotype for the 13 STRs. In 2017, the FBI increased the core STRs from 13 to 20 (65) comprising: CSF1PO, FGA, THO1, TPOX, VWA, D3S1358, D5S818, D7S820, D8S1179, D13S317, D16S539, D18S51, D21S11, D1S1656, D2S441, D2S1338, D10S1248, D12S391, D19S433, D22S1045 and amelogenin. Many countries have since emulated the FBI and compiled CODIS concept and database.

### Mitochondrial DNA Typing

The mtDNA sequence typing used in forensics primarily involves sequencing of the hypervariable (HV) regions, HV1 (positions 16,024–16,365), HV2 (positions 73–340) and HV3 (positions 438–574) (66–71). Direct sequencing is normally performed using the ABI 310/3130/3730/3730xl or next generation sequencer (NGS). Sequencing is done on these instruments using the Sanger’s dideoxy chain termination method. The incorporation of dideoxy nucleotides in newly synthesised DNA strands results in termination of the elongation process (13). The small size and relatively high inter-person variability of the HV regions are very useful features for forensic testing purposes.

**Figure 6:**
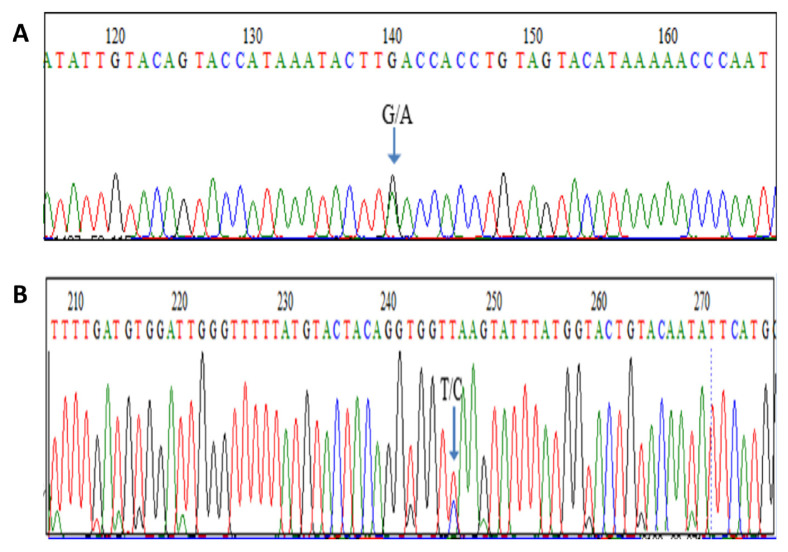
Heteroplasmy in mtDNA. The maternal transmission of mtDNA results in homoplasmic individuals, who typically have a single mtDNA haplotype, the maternal one. Heteroplasmy is the presence of more than one type of mtDNA within an individual. A person is considered as heteroplasmic if she/he carries more than one detectable mtDNA type. Sequence chromatogram showing heteroplasmy in the HVS-I region, at the nucleotide position indicated A): G and A and B): T and C

A person is considered as heteroplasmic (Figures 6A and 6B) if she/he carries more than one detectable mtDNA type. There are two classes of heteroplasmy, related to length polymorphisms and to point substitutions (72, 73). Only the latter is important for forensic human identification. Heteroplasmy manifests itself in diverse ways. An individual may show more than one mtDNA type in a single tissue. An individual may be heteroplasmic in one tissue sample and homoplasmic in another one. Furthermore, an individual may exhibit one mtDNA type in one tissue and a different type in another tissue. Of the three possible scenarios, the last one is the least likely to occur. The mtDNA sequence of siblings and all maternal relatives is identical provided there is no mutation. The shared identical mtDNA in all maternal relatives is known as homoplasmic. However occasional heteroplasmy may be observable in maternal relatives due to mutation.

Heteroplasmy played an important role in identifying the bones of Tsar Nicholas II among nine sets of bones excavated in a mass grave near Yekaterinburg, in 1991. mtDNA sequences from bone of the presumed Tsar matched two living maternal relatives except at a single position, where the bone sample had a mixture of matching (T) and mismatching (C) bases. Cloning experiments indicated that this mixture was due to heteroplasmy within the Tsar. At the request of the Russian Federation government, the skeletal remains of the Tsar’s brother Georgij Romanov was analysed in order to gain further insight into the occurrence and segregation of heteroplasmic mtDNA variants in the Tsar’s maternal lineage. The mtDNA sequence of Georgij Romanov matched that of the putative Tsar and was heteroplasmic at the same position. This confirmed heteroplasmy in the Tsar’s lineage and it was powerful evidence supporting the identification of Tsar Nicholas II (74, 75).

In forensic analysis, both mtDNA sequences of a reference sample and an evidence sample(s) are compared. If the mtDNA sequences are identical, the samples cannot be excluded since they must have the same origin or derive from the same maternal lineage. Similarly, samples cannot be excluded when heteroplasmy is observed at the same nucleotide positions in both samples. Similarly, when one sample is heteroplasmic and the other is homoplasmic but they both share at least one mtDNA species, the samples cannot be excluded since they may have the same origin. It is suggested that samples with mtDNA with one-base difference should be further evaluated, mainly regarding their rate of mutation (39). When two or more nucleotide differences exist between the two sequences, the overall interpretation is exclusion.

mtDNA sequence from an evidence sample and one from a known reference sample cannot be excluded as originating from the same source, it is desirable to convey some information about the mtDNA profile’s rarity. The current practice is to count the number of times a particular sequence is observed in a population. The sequence of HV region from the haploid mtDNA genome is known as haplotype. Similar sequences form a group called haplogroup. Haplotype frequency is a measure of how commonly the sequence is present in a specific population and therefore, is a measure of discriminatory power. Mitochondrial haplotype database in EMPOP and Mitomapare sources for analysing the forensic data. mtDNA analysis is an appropriate method to use for charred remains, degraded specimens, old skeletal and fingernail samples, hair shafts etc. mtDNA is of value in identifying mass disaster victims and in cases where the DNA is degraded or the source of the sample does not contain enough genomic nuclear DNA for analysis (74–76).

### Quality Assurance

DNA typing using STRs is widely accepted as infallible evidence in crime and kinship testing. To provide quality in the standard of analysis and interpretation, a group of scientists was formed by the FBI-Scientific Working Group on DNA Analysis Methods (SWGDAM) (77). This group prescribes certain standards regarding the procedure of carrying out DNA typing and provide guidelines on interpretation of electropherogram results in STR typing. It also prescribes qualification of analysts and their duties. It prescribes for internal and external audit of DNA lab and a prescription for proficiency testing of DNA analysts.

## Conclusion

Autosomal STRs-based DNA profiling has proven as an incredible technique in identification and individualisation of human biospecimens collected from crime scenes. The technique and related statistics for interpretation are robust and highly reproducible. Methods of automation, increasing the speed and output and reliability of STR methods will continue, and continued research may pave way for portable, miniature chips to allow analysis of DNA directly at the crime scene.

## Figures and Tables

**Table 1:** Calculation of RMP in a hypothetical case

STRs tested	Locus					

	vWA		D7S820		TPO X	
Blood stain genotype	14	14	12	15	10	12
Suspect 1 genotype	14	15	12	15	10	11 (suspect 1 excluded)
Suspect 2 genotype	14	14	12	15	10	12
Allele frequency the population	Allele 14 = 0.134		Allele 12 = 0.215	Allele 15 = 0.091	Allele 10 = 0.198	Allele 12 = 0.082
The expected genotype frequency for two alleles in a population in HWE	p^2^ = (0.134)^2^ = 0.018		2pq = 2 × 0.215 × 0.091 = 0.0391		2pq = 2 × 0.198 × 0.082 = 0.0325	

*RMP based on three STRs	0.018 × 0.0391 × 0.0325 = 0.000023; LR = I/0.000023 = 1 in 43,478 individuals					

Notes:

* RMP = random match probability; LR = likelihood ratio

**Table 2:** Calculation of probability of paternity in a hypothetical case

STRs	Mother	Child	Alleged father (AF) – X	Contribution by X (AF)	Random man – Y = allele frequency in the population	*PI
D3S1358	10, 11	10, 12	12 12	1	Allele 12 = 0.185	1/0.185 = 5.405
D5S818	15, 16	11, 16	11 11	1	Allele 11 = 0.053	1/0.053 = 18.868
D21S11	25 26	25, 30	28, 30	0.5	Allele 30 = 0.235	0.5/0.235 = 2.128
**CPI	5.405 × 18.868 × 2.128 = 217.016					
**POP	CPI / CPI +(1 − ****PP) × 100 = 217.016/217.516 × 100 = 99.77					
Probability statement as per ****AABB guidelines	PP = 99.1 to 99.8 = The AF is extremely likely being the biological father of the child. (Note: PP = 99.9 = practically proved that the AF is the biological father of the child)					

Notes:

* PI = paternity index;

** CPI = combined paternity index;

*** POP = probability of paternity;

**** PP = prior probability;

***** AABB = American Association of Blood Banks
